# Thermally stable dielectric responses in uniaxially (001)-oriented CaBi_4_Ti_4_O_15_ nanofilms grown on a Ca_2_Nb_3_O_10_^−^ nanosheet seed layer

**DOI:** 10.1038/srep20713

**Published:** 2016-02-15

**Authors:** Junichi Kimura, Itaru Takuwa, Masaaki Matsushima, Takao Shimizu, Hiroshi Uchida, Takanori Kiguchi, Takahisa Shiraishi, Toyohiko J. Konno, Tatsuo Shibata, Minoru Osada, Takayoshi Sasaki, Hiroshi Funakubo

**Affiliations:** 1 Department of Innovative and Engineered Materials, Tokyo Institute of Technology, 4259 Nagatsuta-cho, Midoriku, Yokohama, Kanagawa 226-8502, Japan; 2 Materials Research Center for Element Strategy, Tokyo Institute of Technology, 4259 Nagatsuta-cho, Midori-ku, Yokohama 226-8503, Japan; 3 Department of Materials and Life Science, Sophia University, Chiyoda, Tokyo, 102-8554, Japan; 4 Institute for Materials Research, Tohoku University, 2-1-1 Katahira, Aoba-ku, Sendai 980-8577, Japan; 5 International Center for Materials Nanoarchitectonics (WPI-MANA), National Institute for Materials Science (NIMS), 1-1 Namiki, Tsukuba, Ibaraki, 305-0044, Japan

## Abstract

To realize a high-temperature capacitor, uniaxially (001)-oriented CaBi_4_Ti_4_O_15_ films with various film thicknesses were prepared on (100)_*c*_SrRuO_3_/Ca_2_Nb_3_O_10_^−^ nanosheet/glass substrates. As the film thickness decreases to 50 nm, the out-of-plane lattice parameters decrease while the in-plane lattice ones increase due to the in-plane tensile strain. However, the relative dielectric constant (*ε*_r_) at room temperature exhibits a negligible degradation as the film thickness decreases to 50 nm, suggesting that *ε*_r_ of (001)-oriented CaBi_4_Ti_4_O_15_ is less sensitive to the residual strain. The capacitance density increases monotonously with decreasing film thickness, reaching a value of 4.5 μF/cm^2^ for a 50-nm-thick nanofilm, and is stable against temperature changes from room temperature to 400 °C irrespective of film thickness. This behaviour differs from that of the widely investigated perovskite-structured dielectrics. These results show that (001)-oriented CaBi_4_Ti_4_O_15_ films derived using Ca_2_Nb_3_O_10_^−^ nanosheets as seed layers can be made candidates for high-temperature capacitor applications by a small change in the dielectric properties against film thickness and temperature variations.

The demand is increasing for capacitors that can function at elevated temperatures in electronic circuits in automobiles, power devices, and light-emitting diodes^1^,^2^,^3^,^4^. One of the most promising candidates is a ceramic capacitor due to its thermal stability. Recently, various approaches have been used to improve the performance of ceramic capacitors, including the preparation of thin-film capacitors composed of inorganic dielectrics such as perovskite-structured oxides. Although (Ba, Sr)TiO_3_-based dielectrics, which have a high dielectric constant (*ε*_r_) near room temperature, are the most widely investigated ceramic capacitor materials^5^,^6^,^7^, they are not suitable for high-temperature capacitor applications due to their temperature instability within the required temperature range (i.e., *ε*_r_ changes drastically around the phase transition temperature). Consequently, they cannot achieve high *ε*_r_ and a small-temperature dependence of the capacitance simultaneously due to the trade-off relationship^5^,^7^.

In addition, the *ε*_r_ of thin-film capacitors produced using (Ba, Sr)TiO_3_-based materials as the dielectric layer is severely degraded as the film thickness decreases, making it difficult to enhance the capacitance density by decreasing the film thickness^8^,^9^,^10^. One possible reason for this sensitivity to film thickness may be the high strain sensitivity of *ε*_r_, which remains as the film thickness decreases. These phenomena are common material problems for conventional perovskite dielectrics, suggesting that another novel capacitor material is necessary.

To realize novel dielectrics with a large capacitance density and small change in *ε*_r_ at elevated temperatures, we previously proposed bismuth layer-structured dielectrics (BLSDs)^11^. BLSDs have a natural superlattice structure along the *c*-axis [(001)-orientation], which consists of two kinds of two-dimensional monolayers (i.e., a bismuth oxide (Bi_2_O_2_)^2+^ layer and a pseudoperovskite layer generally described as (A_*m*−1_B_*m*_O_3*m*+1_)^2−^, where *m* is the number of BO_6_ octahedra in the pseudoperovskite layer)^12^,^13^. Compared to BaTiO_3_-based materials, some BLSDs exhibit a high Curie temperature and their *ε*_r_ shows small temperature dependence along the stack direction (i.e., the *c*-axis) up to the high temperature region^14^,^15^,^16^. These materials also have important additional features in which the degradation in *ε*_r_ as the film thickness decreases is small (i.e., a small “size effect”), allowing a high capacitance density to be realized^17^. This behaviour showing the small strain sensitivity of *ε*_r_, which is attributed to the structural two-dimensionality of BLSDs, differs from conventional perovskite-based dielectrics.

We have already investigated the dielectric properties of 500-nm-thick (001)-oriented epitaxial films of CaBi_4_Ti_4_O_15_, which are BLSDs prepared on (100)_*c*_SrRuO_3_/(100)SrTiO_3_. These films not only have an *ε*_r_ of about 190 at room temperature, but they also display a stable capacitance against the applied electric field as well as temperature variations up to 500 °C^11^. Moreover, the *ε*_r_ for epitaxial thin films of SrBi_4_Ti_4_O_15,_ which is another BLSD, does not drastically degrade as the film thickness decreases to 15 nm^17^. These reports clearly show that the *ε*_r_ of thin-film capacitors of epitaxial BLSDs have a superior temperature-dependent performance compared to BaTiO_3_-based ones and may overcome issues observed in conventional BaTiO_3_-based materials in high-temperature applications.

However, the dielectric properties of BLSDs include a strong anisotropy due to the crystal structure of BLSDs^16^. In fact, our group has previously reported that the dependence of the film thickness on the dielectric property in BLSDs is strongly influenced by the tilting angle of the *c*-axis from the substrate normal^18^. Therefore, reproducible growth of (001)-oriented BLSDs films (i.e., the stack direction of the layers) is a critical issue to achieve a stable *ε*_r_ as both the film thickness and temperature decrease.

From the viewpoint of practical applications, reproducible fabrication of uniaxially (001)-oriented BLSDs films on various common substrates, including amorphous glasses and (100) Si wafers, is a critical issue for practical applications even if the in-plane orientation of these films is more random than that of epitaxial films. Shibata *et al.* reported the {100} orientation control of SrTiO_3_ films on amorphous glass using a seed layer of Ca_2_Nb_3_O_10_^−^ nanosheets^19^. Based on their work, we envisioned that a Ca_2_Nb_3_O_10_^−^ seed layer could control the (001) orientation of BLSD films because (001)-oriented BLSD films were grown on (100)SrTiO_3_ single crystals^15^.

In the present study, we sought to enhance the capacitance density of Pt/(001)-oriented CaBi_4_Ti_4_O_15_/(100)_*c*_SrRuO_3_ capacitors by decreasing the film thickness of the CaBi_4_Ti_4_O_15_ layer. This should allow the capacitor size to be scaled down or the total capacitance to be increased. In the case of (Ba, Sr)TiO_3_ film capacitors, *ε*_r_ is reported to change drastically not only with film thickness but also with temperature, which makes it difficult to design a capacitor with a small capacitance change over a wide temperature range^8^,^9^,^10^. For (001)-oriented CaBi_4_Ti_4_O_15_ films, the capacitance density reaches 4.5 μF/cm^2^ as the film thickness is decreased to 50 nm, and the capacitance has a small temperature coefficient from room temperature to 400 °C despite the increase in the residual strain as the film thickness decreases. The present results indicate that nanosheet-buffered uniaxially (001)-oriented CaBi_4_Ti_4_O_15_ thin films are promising alternatives to (Ba, Sr)TiO_3_-based films in high temperature capacitors.

## Results and discussion

### Crystal structure and microstructure

**Figure 1:**
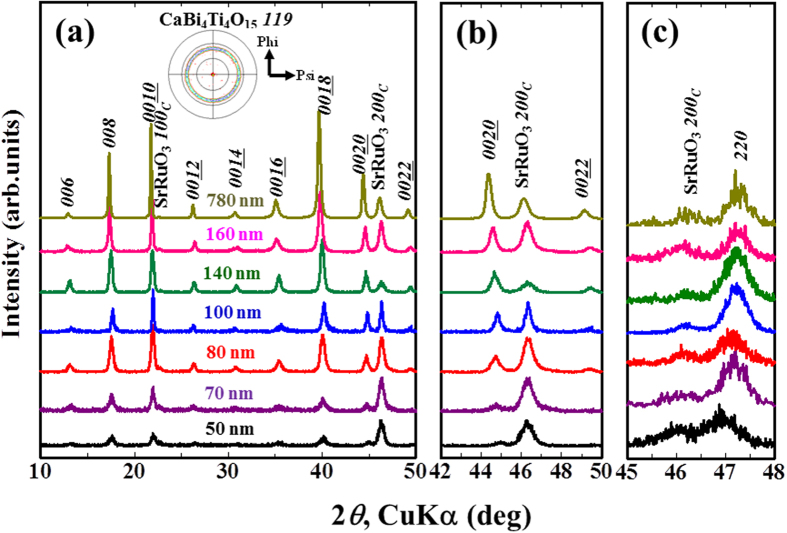
Out-of-plane XRD *θ*–2*θ* patterns of the films with various thicknesses in the region of (**a**) 2*θ* = 10–50° and (**b**) 2*θ* = 42–50° along with (**c**) the in-plane XRD *θ*–2*θ* patterns of the films in the region of 2*θ* = 45–48°. The inset in (**a**) shows the X-ray pole measured at a fixed 2*θ* angle corresponding to CaBi_4_Ti_4_O_15_ *119* (2*θ* = 30.6°) for 780-nm-thick films.

The out-of-plane XRD *θ*–2*θ* patterns for CaBi_4_Ti_4_O_15_ films with various thicknesses show only the *h00*_*c*_ and *00 l* diffraction peaks of SrRuO_3_ and CaBi_4_Ti_4_O_15_ (Fig. 1(a)), respectively, suggesting a (001) single orientation of CaBi_4_Ti_4_O_15_ regardless of the film thickness. The X-ray pole figure measurement at a fixed 2*θ* angle corresponding to CaBi_4_Ti_4_O_15_ *119* (2*θ* = 30.6°) shows strong ring-shape-peaks at *Psi* ≑ 50° for 780-nm-thick films (inset of Fig. 1(a)), indicating a uniaxial (001)-orientation along the substrate surface normal with an in-plane random orientation.

Figure 1(b) presents the enlarged XRD patterns around the CaBi_4_Ti_4_O_15_ *0020* peak in Fig. 1(a). The diffraction angles of the CaBi_4_Ti_4_O_15_ *0020* peak become higher as the film thickness decreases, indicating that the *c*-axis lattice parameters along the substrate normal decrease. Moreover, the in-plane XRD patterns around CaBi_4_Ti_4_O_15_ *220* are shown in Fig. 1(c). The CaBi_4_Ti_4_O_15_ *220* peaks shift toward a lower diffraction angle with decreasing film thickness, suggesting an increase in the in-plane lattice parameter (i.e., the *a*(*b*)-axis).

**Figure 2:**
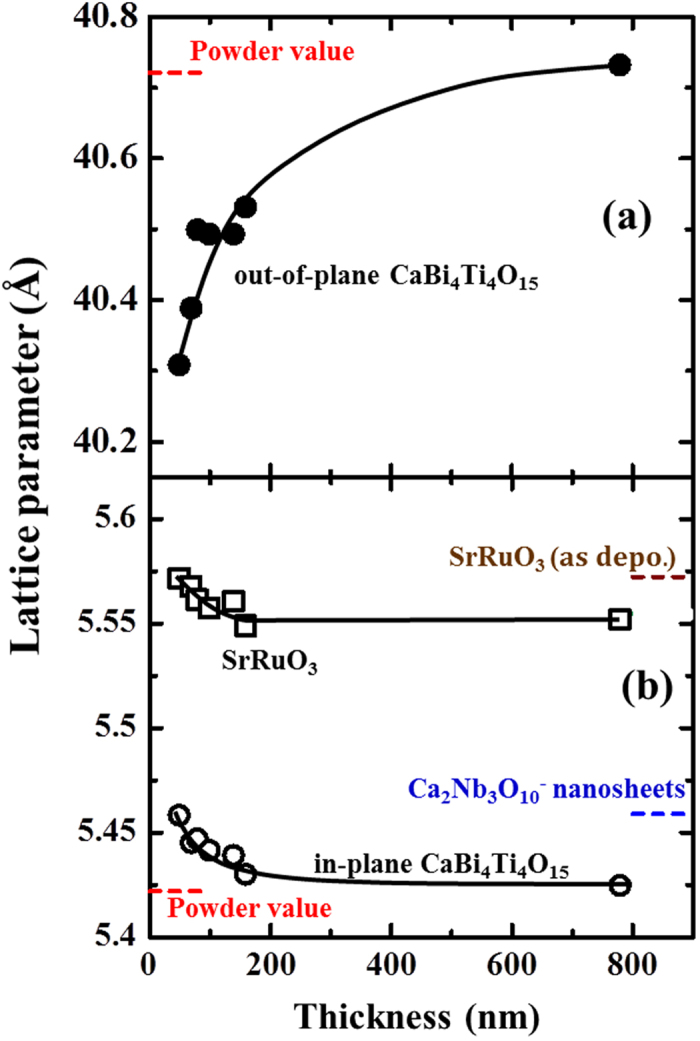
(**a**) Out-of-plane and (**b**) in-plane lattice parameters of the films as functions of film thickness. (●: out-of-plane; ○: in-plane). (**b**) A plot of the in-plane lattice parameter of the SrRuO_3_ layer (□) determined from the data in Fig. 1(c) is shown. Reference data of Ca_2_Nb_3_O_10_^19^,^20^ are also shown.

Figure 2(a,b) plot the out-of-plane *c*-axis and in-plane *a*(*b*)-axis lattice parameters calculated from the diffraction data shown in Fig. 1(a–c) as functions of film thickness, respectively. Additionally, reference data for the CaBi_4_Ti_4_O_15_ powder are shown^14^. The in-plane XRD measurements confirm that clear peak splitting is not detected for the *200*/*020* peak irrespective of the film thickness because the lattice parameters of the *a*- and *b*-axes are very close in the CaBi_4_Ti_4_O_15_ crystal lattice (data not shown). The out-of-plane and in-plane lattice parameters for the 780-nm-thick film are almost the same as the reported data for strain-free CaBi_4_Ti_4_O_15_ powder. As the film thickness decreases, the out-of-plane *c*-axis lattice parameter decreases and drops drastically below 200 nm, whereas the in-plane *a*-axis and *b*-axis lattice parameters increase from 5.42 to 5.46 Å.

To analyse the change in the lattice parameter of CaBi_4_Ti_4_O_15_ films, Fig. 2(b) also plots the in-plane lattice parameter of the underlying SrRuO_3_ bottom electrodes obtained from Fig. 1(c) as well as the in-plane lattice parameter of the as-deposited SrRuO_3_ layer before CaBi_4_Ti_4_O_15_ film deposition along with the reference data of Ca_2_Nb_3_O_10_^−^20. The in-plane lattice parameter of CaBi_4_Ti_4_O_15_ for a 780-nm-thick film is almost equivalent to that of the CaBi_4_Ti_4_O_15_ powder, suggesting that the crystal lattice of the 780-nm-thick CaBi_4_Ti_4_O_15_ film is almost relaxed. Meanwhile, the in-plane lattice parameter of CaBi_4_Ti_4_O_15_ increases as the film thickness decreases. A similar trend is observed for the in-plane lattice parameters of SrRuO_3_. It should be noted that the in-plane lattice parameter of SrRuO_3_ is almost identical to that of the as-deposited 50-nm-thick SrRuO_3_ films before CaBi_4_Ti_4_O_15_ deposition but not to that of the Ca_2_Nb_3_O_10_^−^ nanosheets (Fig. 2(b)) because the crystal lattice of the as-deposited SrRuO_3_ layer is not fully clamped on the lattice of the Ca_2_Nb_3_O_10_^−^ nanosheets. CaBi_4_Ti_4_O_15_ also possesses a different in-plane lattice parameter from those of the SrRuO_3_ and Ca_2_Nb_3_O_10_ nanosheets, indicating that the change in the lattice parameter of CaBi_4_Ti_4_O_15_ films is not perfectly clamped by the crystal lattice of the underlying SrRuO_3_ electrode layer. The increase in the in-plane lattice parameter of the CaBi_4_Ti_4_O_15_ film (Fig. 2(b)) suggests that the in-plane residual strain of CaBi_4_Ti_4_O_15_ films increases as the film thickness decreases. The large in-plane lattice parameter of thin CaBi_4_Ti_4_O_15_ is considered to be a response to the larger lattice parameter of the underlying SrRuO_3_ layer, because the CaBi_4_Ti_4_O_15_ films are thinner than the 50 nm-thick SrRuO_3_ layer. Here, as-deposited SrRuO_3_ films have a larger unit cell than SrRuO_3_ powders, i.e., a larger in-plane strain, due to the damage occurring under the sputtering process using the present deposition conditions, as reported previously^21^,^22^. The in-plane lattice parameter of CaBi_4_Ti_4_O_15_ relaxed and approached the bulk value together with the SrRuO_3_ layer with the increase in the total thickness.

**Figure 3:**
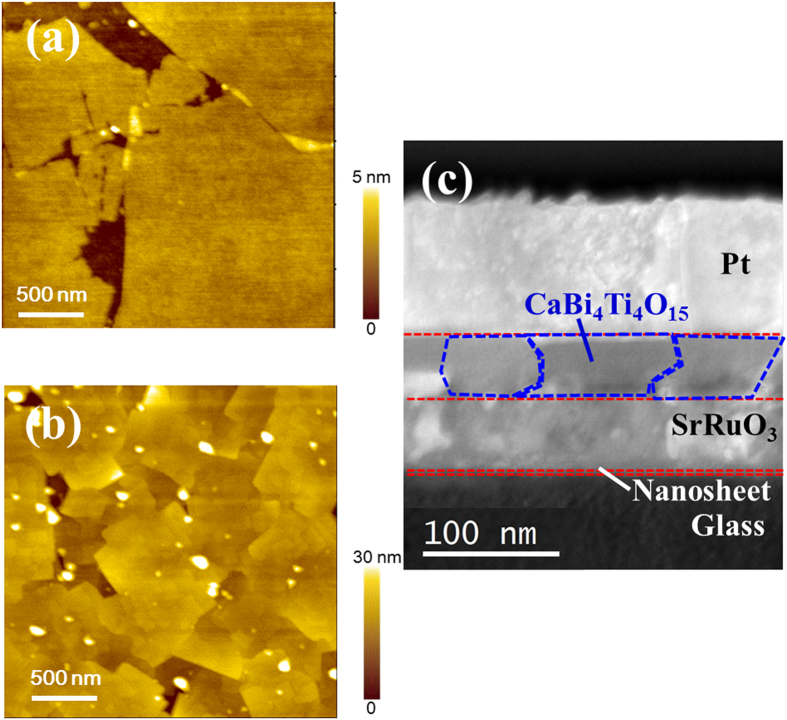
Atomic force microscopy (*AFM*) topographic images of (**a**) Ca_2_Nb_3_O_10_ nanosheets on glass substrates and (**b**) CaBi_4_Ti_4_O_15_ films, together with (**c**) cross sectional low-angle annular dark field-scanning transmission electron microscope (LAADF-STEM) images of the films.

Figure 3(a,b) shows atomic force microscopy (*AFM*) topographic images of Ca_2_Nb_3_O_10_ nanosheets on glass substrates and CaBi_4_Ti_4_O_15_ films. Ca_2_Nb_3_O_10_ nanosheets have a flat surface with 1–2 units in thickness and 3–10 μm in-plane width. On the other hand, the lateral grain sizes of CaBi_4_Ti_4_O_15_ films are about 100 nm, which is smaller than the grain sizes of Ca_2_Nb_3_O_10_ nanosheets. Figure 3(c) shows the cross sectional low-angle annular dark field-scanning transmission electron microscope (LAADF-STEM) image of the films. Dense CaBi_4_Ti_4_O_15_ films are found to be deposited and their grain sizes look almost identical to those from *AFM* observations.

### Dielectric property

**Figure 4:**
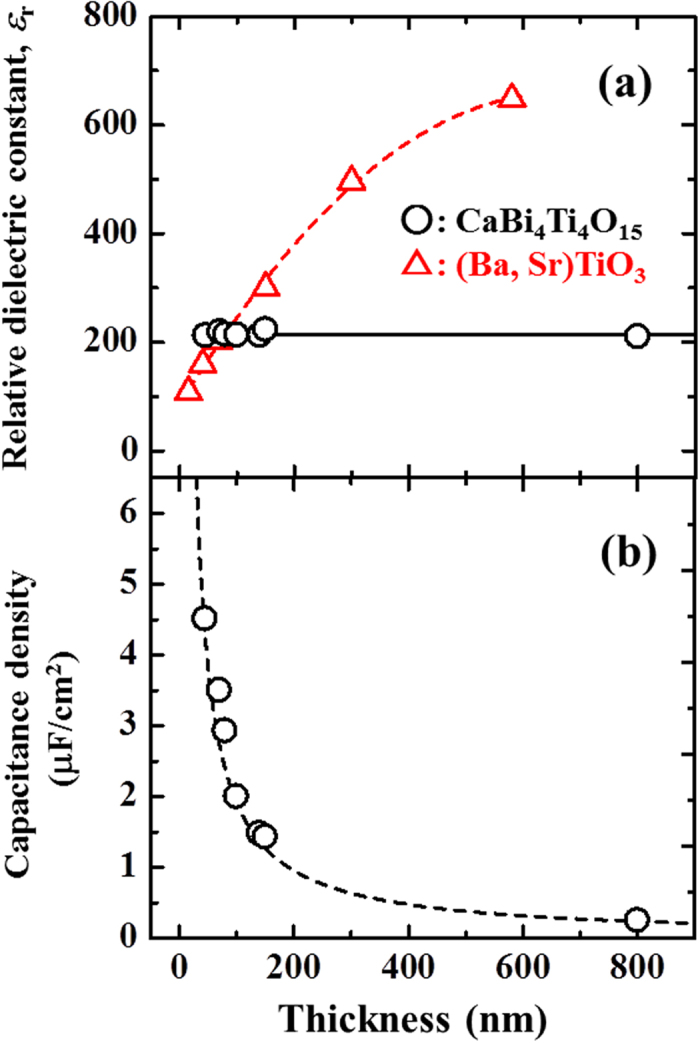
(**a**) Relative dielectric constant (*ε*_*r*_) and (**b**) capacitance density measured at room temperature as functions of film thickness. (○: CaBi_4_Ti_4_O_15_; △: (Ba_0.7_ Sr_0.3_)TiO_3_ in ref. 9).

Figure 4(a) shows the *ε*_r_ for the (001)-oriented CaBi_4_Ti_4_O_15_ films measured at room temperature and 100 kHz as functions of the film thickness. The *ε*_r_ of these films is approximately 210, which is a negligible degradation as the film thickness decreases to 50 nm. This is almost equivalent to the results for epitaxial films^17^. As a reference of *ε*_r_, the *ε*_r_ values for (Ba_0.7_ Sr_0.3_)TiO_3_ films reported by Parker *et al.,* which continuously degrade as the film thickness decreases below 600 nm, are also plotted in Fig. 4(a) 9. The *ε*_r_ value of (Ba_0.7_ Sr_0.3_)TiO_3_ films becomes smaller than that of CaBi_4_Ti_4_O_15_ with a film thickness below 80 nm, suggesting that a CaBi_4_Ti_4_O_15_ layer less than 80-nm thick achieves a thin-film capacitor with a higher capacitance density than the conventional (Ba_0.7_ Sr_0.3_)TiO_3_ one.

Figure 4(b) shows the capacitance density at room temperature as a function of film thickness, where the dashed line indicates the estimated data assuming that the CaBi_4_Ti_4_O_15_ film has an almost constant *ε*_r_ value (*ε*_r_ = 210, which is equivalent with that of the 780-nm-thick specimen) as the film thickness changes. The theoretical capacitance density is generally proportional to the inverse of the film thickness. The capacitance densities of the obtained (001)-oriented CaBi_4_Ti_4_O_15_ films are almost identical to the estimated values. As the film thickness decreases, the density increases and reaches a value of 4.5 μF/cm^2^ at a thickness of 50 nm.

It is noteworthy that *ε*_r_ remains almost constant despite the increase in the residual strain as the thickness is scaled down to 50-nm (Fig. 2). Thus, we confirm that *ε*_r_ of the (001)-oriented CaBi_4_Ti_4_O_15_ films is free from the size-effect of the capacitance. This is an important feature not only for epitaxial (001)-oriented BLSD films, as already mentioned, but also for uniaxially (001)-oriented ones.

**Figure 5:**
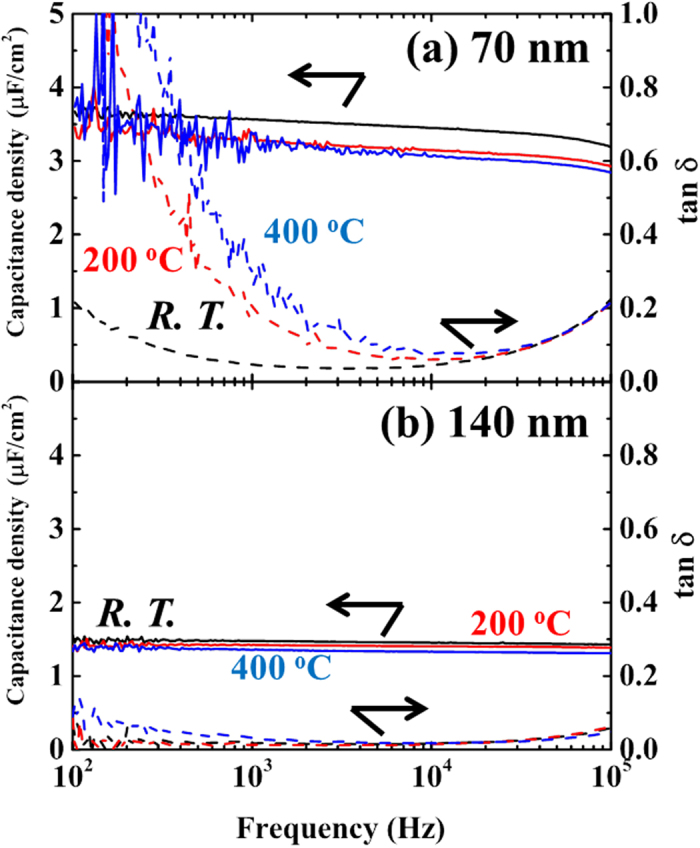
Frequency dependencies of the capacitance density and dielectric loss, tan δ, measured from room temperature to 400 °C for (**a**) 70 and (**b**) 140 nm-thick CaBi_4_Ti_4_O_15_ films.

Figure 5 shows the frequency dependencies of the capacitance density and dielectric loss, tan δ, measured from room temperature to 400 °C for 70 and 140 nm-thick CaBi_4_Ti_4_O_15_ films. The capacitance density is almost independent of the measurement frequency from 100 to 10 kHz irrespective of the measurement temperature up to 400 °C. On the other hand, tan δ decreases with increasing measurement frequency from 100 to 10 kHz, but increases again above 10 kHz for 70 nm thick films as shown in Fig. 5(a). The relatively large dielectric loss at low frequency may originate from the leakage of the capacitor. The tan δ value increases as the measurement temperature increases due to the contribution from leakage current. However, the tan δ of 140 nm thick films remains at a low value below 4%, almost independent of the frequency from 10 to 10 kHz, even at the highest measurement temperature of 400 °C, as shown in Fig. 5.

It must be mentioned that no noticeable peel-off of CaBi_4_Ti_4_O_15_ films was detected, either in the case of as-deposited films or in the case of films at the highest measurement temperatures of up to 400 °C irrespective of the film thickness.

**Figure 6:**
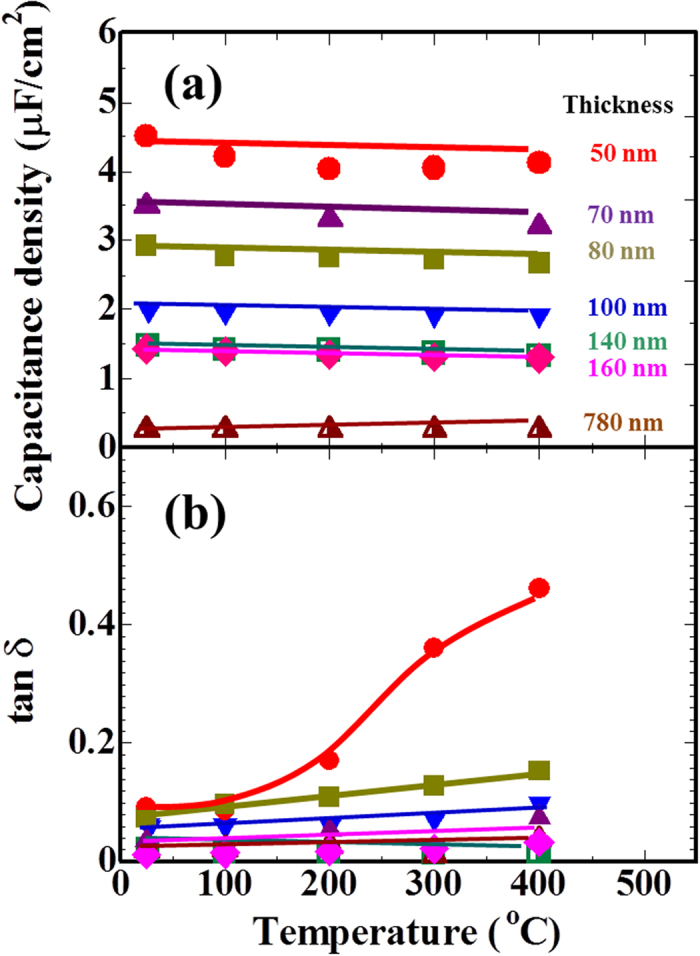
Capacitance density (**a**) and dielectric loss (tan δ) (**b**) of the films as a function of temperature.

Figure 6 plots the temperature dependence of the capacitance density and tan δ for (001)-oriented CaBi_4_Ti_4_O_15_ films with different thicknesses. The capacitance density of these films has a negative slope versus temperature, but shows a small temperature coefficient of capacitance (*TCC, TCC* ≡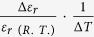, which is based on the definition of the Electronic Industries Alliance) in the temperature range from 25 to 400 °C regardless of film thickness down to 50 nm^23^. These *TCC* values fall within the range from −350 to −120 ppm/°C; this characteristic is almost equivalent to that of the epitaxial films in our previous work^11^. These results imply that uniaxially (001)-oriented CaBi_4_Ti_4_O_15_ films with an in-plane random crystal orientation have dielectric and insulating properties with a small temperature dependence similar to epitaxial films. The capacitance density increases with decreasing film thickness for all temperature regions (Fig. 6(a)). On the other hand, the tan δ value increases with the temperature due to the increase of the leakage current, as shown in Fig. 6(b). However, it decreases with increasing film thickness, especially if it is below 10% up to 400 °C for the films above 100 nm in thickness.

**Figure 7:**
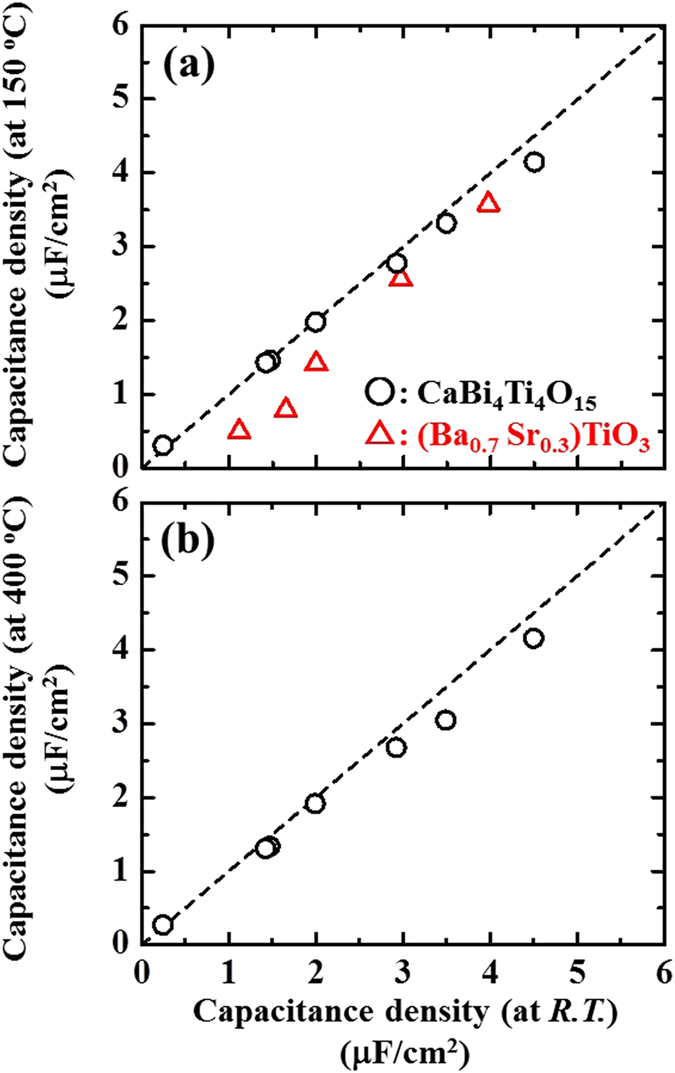
Relationships between the capacitance densities for the films measured at room temperature and (**a**) 150 °C and (**b**) 400 °C. (○: CaBi_4_Ti_4_O_15_; △: (Ba_0.7_ Sr_0.3_)TiO_3_ in ref. 9).

To analyse the effect of thickness on the capacitance density at elevated temperatures, Fig. 7(a) compares the capacitance densities for (001)-oriented CaBi_4_Ti_4_O_15_ films measured between 150 °C and room temperature. The dashed line indicates the case without a degraded capacitance density up to 150 °C. The measured data are almost located on the dashed line, indicating a negligible difference in the capacitance change between these two temperatures. Figure 7(a) also plots the data for (Ba_0.7_ Sr_0.3_)TiO_3_ films as a reference; as the temperature increases to 150 °C, the capacitance density for thicker films drastically decreases and has a negative *TCC* value^9^. In addition, Fig. 7(b) compares the capacitance densities at 400 °C and room temperature. The measured data are also located for (001)-oriented CaBi_4_Ti_4_O_15_ films on the dashed line even at temperatures as high as 400 °C. The estimated degradations of the capacitance density from room temperature to 150 and 400 °C are 7% and 9% for 50-nm-thick CaBi_4_Ti_4_O_15_ films, respectively. Unlike conventionally investigated (Ba_0.7_ Sr_0.3_)TiO_3_ films where the capacitance decreases to 55% between room temperature and 150 °C, the capacitance density for uniaxially (001)-oriented CaBi_4_Ti_4_O_15_ films shows a small degradation as the temperature increases to 400 °C. These results suggest that (001)-oriented CaBi_4_Ti_4_O_15_ films produced using Ca_2_Nb_3_O_10_^−^ nanosheet seed layers are novel candidates for high-temperature adaptive capacitor applications due to the superior temperature stability of their electric properties up to 400 °C along with their high capacitance density, which is derived from the small “size effect” upon scaling down the film thickness.

### Summary

Uniaxially (001)-oriented CaBi_4_Ti_4_O_15_ films with various film thicknesses were prepared on (100)_*c*_SrRuO_3_/Ca_2_Nb_3_O_10_^−^ nanosheets/glass substrates. All films exhibited a (001) single orientation along the substrate surface normal, but had a random in-plane orientation. The continuous increase in the residual tensile strain as the film thickness decreases leads to a reduction in the out-of-plane “*c*-axis” lattice parameters and an increase in the in-plane “*a*- and *b*-axes” ones. However, the change in *ε*_r_ is unremarkable as the film thickness decreases to 50 nm. Consequently, the monotonous increase in the capacitance density is proportional to the inverse of the film thickness. The capacitance densities of the obtained films show small *TCC* values up to a temperature of 400 °C and are almost independent of film thickness between 50 and 780 nm. By contrast, conventional (Ba, Sr)TiO_3_-based films do not exhibit these behaviours. These results indicate that uniaxially (001)-oriented CaBi_4_Ti_4_O_15_ thin films prepared on Ca_2_Nb_3_O_10_^−^ nanosheet–buffered substrates have potential in high-temperature capacitor applications.

## Methods

Uniaxially (001)-oriented CaBi_4_Ti_4_O_15_ films with various film thicknesses were prepared on (100)_*c*_SrRuO_3_/(Ca_2_Nb_3_O_10_^−^ nanosheets)/glass substrates. The CaBi_4_Ti_4_O_15_ and about 50 nm-thick SrRuO_3_ layers were fabricated on (Ca_2_Nb_3_O_10_^−^ nanosheets)/glass (Corning#1737) by the RF-magnetron sputtering method at substrate temperatures of 600 and 550 °C, respectively. The 1–2 unit-thick Ca_2_Nb_3_O_10_^−^ nanosheet layers were coated onto glass substrates by the Langmuir-Blodgett process^20^,^24^,^25^. We chose CaBi_4_Ti_4_O_15_ as the dielectric layer because its high Curie temperature of 790 °C enables a small temperature dependence of the capacitance at elevated temperatures. The deposition time controlled the CaBi_4_Ti_4_O_15_ film thickness between 50 and 780 nm. Details of the deposition are described elsewhere^11^. After preparing circular Pt top electrodes (100 μm*ϕ*) by electron-beam deposition, the Pt/CaBi_4_Ti_4_O_15_/SrRuO_3_ capacitors were annealed at 400 °C for 30 min under O_2_ gas flow.

The constituent phase and crystal orientation of the deposited films were identified by X-ray diffraction (XRD) using a Philips X’pert MRD with Cu *K*α radiation. The residual strain state was also estimated by in-plane XRD measurements using a Rigaku Smart-lab diffractometer with Cu *K*α radiation. The electrical properties under various temperatures were measured using an impedance analyser (HP4194A, Agilent) and a sample-heating stage.

## Additional Information

**How to cite this article**: Kimura, J. *et al.* Thermally stable dielectric responses in uniaxially (001)-oriented CaBi_4_Ti_4_O_15_ nanofilms grown on a Ca_2_Nb_3_O_10_^-^ nanosheet seed layer. *Sci. Rep.* **6**, 20713; doi: 10.1038/srep20713 (2016).
